# Long-term impact of infant immunization on hepatitis B prevalence: a systematic review and meta-analysis

**DOI:** 10.2471/BLT.17.205153

**Published:** 2018-05-14

**Authors:** Kate Whitford, Bette Liu, Joanne Micallef, J Kevin Yin, Kristine Macartney, Pierre Van Damme, John M Kaldor

**Affiliations:** a Kirby Institute, Level 6, Wallace Wurth Building, UNSW Sydney, Kensington, Sydney, 2052 Australia.; b School of Public Health and Community Medicine, UNSW Sydney, Sydney, Australia.; c Sydney School of Public Health, The University of Sydney, Sydney, Australia.; d National Centre for Immunisation, Research and Surveillance, Sydney, Australia.; e Centre for the Evaluation of Vaccination, University of Antwerp, Antwerp, Belgium.

## Abstract

**Objective:**

To conduct a systematic review and meta-analysis of the long-term impact of infant vaccination on the prevalence of hepatitis B virus (HBV) infection at the population level.

**Methods:**

We searched online databases for articles reporting comparisons between population cohorts aged ≥ 15 years who were exposed or unexposed to infant HBV immunization programmes. We categorized programmes as universal or targeted to infants whose mothers were positive for hepatitis B surface antigen (HBsAg). We included studies reporting prevalence of hepatitis B core antibody (HBcAb), HBsAg, or both. We evaluated the quality of the study methods and estimated the relative reduction in the prevalence of infection.

**Findings:**

Of 26 studies that met the inclusion criteria, most were from China (20 studies). The prevalence of HBV infection in unvaccinated and universally vaccinated cohorts ranged from 0.6% (116 of 20 305 people) to 16.3% (60/367) and from 0.3% (1/300) to 8.5% (73/857), respectively. Comparing cohorts with universal vaccination to those without vaccination, relative prevalences were 0.24 (95% confidence interval, CI: 0.16–0.35) for HBsAg and 0.23 (95% CI: 0.17–0.32) for HBcAb. For populations with targeted vaccination, relative prevalences were 0.32 (95% CI: 0.24–0.43) and 0.33 (95% CI: 0.23–0.45), respectively.

**Conclusion:**

The residual burden of infection in cohorts offered vaccination suggests that longer-term evaluations of vaccination coverage, timeliness and other aspects of programme quality are needed. As HBV-vaccinated infant cohorts reach adulthood, ongoing analysis of prevalence in adolescents and young adults will ensure that elimination efforts are on track.

## Résumé

**Objectif:**

Réaliser une revue systématique et une méta-analyse de l'impact à long terme de la vaccination des nourrissons sur la prévalence de l'infection par le virus de l'hépatite B au niveau de la population.

**Méthodes:**

Nous avons consulté des bases de données en ligne à la recherche d'articles établissant des comparaisons entre des cohortes de populations âgées de 15 ans ou plus ayant bénéficié ou non de programmes de vaccination contre le virus de l'hépatite B pour les nourrissons. Nous avons divisé ces programmes en deux catégories: les programmes universels et les programmes ciblés sur des nourrissons dont les mères étaient positives pour l'antigène de surface de l'hépatite B (HBsAg). Nous avons inclus des études indiquant la prévalence de l'état de l'anticorps nucléocapsidique de l'hépatite B (HBcAb), du HBsAg ou des deux. Nous avons évalué la qualité des méthodes d'étude et estimé la diminution relative de la prévalence de l'infection.

**Résultats:**

Sur les 26 études réunissant les critères d'inclusion, 20 provenaient de Chine. La prévalence de l'infection par le virus de l'hépatite B variait de 0,6% (116 individus sur 20 305) à 16,3% (60/367) chez les cohortes non vaccinées, et de 0,3% (1/300) à 8,5% (73/857) chez les cohortes ayant bénéficié d'une vaccination universelle. La comparaison des cohortes ayant bénéficié d'une vaccination universelle avec les cohortes n'ayant reçu aucune vaccination a révélé une prévalence relative de 0,24 (intervalle de confiance, IC, à 95%: 0,16-0,35) pour le HBsAg et de 0,23 (IC à 95%: 0,17-0,32) pour le HBcAb. Dans le cas des populations ayant reçu une vaccination ciblée, la prévalence relative était de 0,32 (IC à 95%: 0,24-0,43) pour le HBsAg et de 0,33 (IC à 95 %: 0,23-0,45) pour le HBcAb.

**Conclusion:**

La charge résiduelle liée à l'infection dans le cas de plusieurs cohortes ayant bénéficié d'une vaccination laisse entendre que des évaluations à long terme de la couverture vaccinale, du caractère opportun et d'autres aspects de la qualité des programmes sont nécessaires. À mesure que les cohortes de nourrissons vaccinés contre le virus de l'hépatite B atteignent l'âge adulte, une analyse continue de la prévalence chez les adolescents et les jeunes adultes permettra de garantir que les efforts d'élimination sont sur la bonne voie.****

## Resumen

**Objetivo:**

Realizar una revisión sistemática y un meta-análisis del impacto a largo plazo de la vacunación infantil sobre la prevalencia de la infección por el virus de la hepatitis B (VHB) a nivel de la población.

**Métodos:**

Investigamos en bases de datos en Internet en busca de artículos que informaran sobre comparaciones entre cohortes de población con edades de ≥ 15 años, que se vieron expuestas o no a programas de inmunización infantil del VHB. Clasificamos los programas como universales o dirigidos a menores de edad, cuyas madres dieron positivo en el antígeno de superficie de la hepatitis B (HBsAg). Incluimos estudios que informan de la prevalencia del anticuerpo core de la hepatitis B (HBcAb) o del estado de HBsAg o de ambos. Evaluamos la calidad de los métodos de estudio y estimamos la reducción relativa en la prevalencia de la infección.

**Resultados:**

De los 26 estudios que cumplían con los criterios de inclusión, la mayoría procedían de China (20 estudios). La prevalencia de la infección por VHB en cohortes sin vacunar y vacunadas de forma universal variaba desde el 0,6% (116 de 20 305 personas) hasta el 16,3% (60/367) y desde el 0,3% (1/300) hasta el 8,5% (73/857), respectivamente. Al comparar las cohortes con vacunación universal y aquellas sin vacunación, la prevalencia relativa fue del 0,24 (intervalo de confianza del 95%, IC: 0,16–0,35) para HBsAg y 0,23 (95% IC: 0,17–0,32) para HBcAb. En aquellas poblaciones con vacunación dirigida, la prevalencia relativa fue del 0,32 (95% IC: 0,24–0,43) y del 0,33 (95% IC: 0,23–0,45), respectivamente.

**Conclusión:**

La carga residual de la infección que ofrece la vacunación en diversas cohortes, sugiere que son necesarias las evaluaciones a largo plazo de la cobertura de vacunación, del carácter oportuno y de otros aspectos sobre la calidad del programa. A medida que las cohortes de niños vacunados contra el VHB alcanzan la edad adulta, un análisis continuo de la prevalencia en adolescentes y en jóvenes asegurará que los esfuerzos para su eliminación van por buen camino.

## ملخص

**الهدف:**

إجراء مراجعة منهجية وتحليل تلوي للتأثير طويل الأجل لتطعيم الرضع، على مدى انتشار عدوى فيروس التهاب الكبد (ب) (HBV) على مستوى السكان.

**الطريقة:**

لقد قمنا بالبحث في قواعد البيانات على الإنترنت عن المقالات التي تعقد مقارنات بين المجموعات السكانية الذين تصل أعمارهم إلى 15 سنة أو أكثر، والذين تعرضوا أو لم يتعرضوا لبرامج تمنيع الرضع ضد فيروس التهاب الكبد (HBV). قمنا بتصنيف البرامج على أنها شاملة، أو مستهدفة للرضع الذين كانت أمهاتهم موجبة لمستضد التهاب الكبد (ب) السطحي (HBsAg). كما قمنا بتضمين الدراسات التي تكشف عن انتشار الأجسام المضادة الأساسية لالتهاب الكبد (ب) (HBcAb)، أو حالة HBsAg، أو كليهما. قمنا بتقييم جودة طرق الدراسة، وتقدير الانخفاض النسبي في انتشار العدوى.

**النتائج:**

من بين 26 دراسة كانت تلبي معايير الاختيار، كانت أغلب الدراسات من الصين (20 دراسة). تراوحت نسبة انتشار عدوى فيروس التهاب الكبد (HBV) في المجموعات غير المحصنة ، من 0.6٪ (116 من 20305 أشخاص) إلى 16.3٪ (60/367)، أما في المجموعات المحصنة عالميا فتراوحت من 0.3٪ (1/300) إلى 8.5٪ (73/857)، على التوالي. وبمقارنة مجموعات التطعيم الشامل بالمجموعات التي لم تحصل على التطعيم، كان الانتشار النسبي 0.24 (فاصل الثقة 95٪ ، من 0.16 إلى 0.35) بالنسبة لـ HBsAg، بينما كان 0.23 (فاصل الثقة 95٪: 0.17 إلى 0.32) بالنسبة لـ HBcAb. أما بالنسبة للسكان في حالة التحصين المستهدف، فقد كان الانتشار النسبي 0.32 (فاصل الثقة 95٪: من 0.24 إلى 0.43)، و0.33 (فاصل الثقة 95٪: 0.23 إلى 0.45) على الترتيب.

**الاستنتاج:**

إن عبء العدوى المتبقي في عدة مجموعات حصلت على التحصين، يشير إلى الحاجة لإجراء تقييمات ذات أجل أطول تشمل تغطية التحصين، والتوقيت، وغيرها من الجوانب الأخرى لجودة البرنامج. ومع وصول مجموعات الرضع التي حصلت على التحصين ضد فيروس التهاب الكبد إلى مرحلة البلوغ، سيضمن التحليل المستمر لانتشار العدوى بين المراهقين والشباب البالغين، أن جهود القضاء على هذه العدوى على المسار الصحيح.

## 摘要

**目的:**

对婴儿免疫接种在群体水平上对乙肝病毒 (HBV) 感染患病率的长期影响进行系统综述和荟萃分析。

**方法:**

我们搜索了线上数据库报道曾接触和未接触婴儿乙肝病毒免疫接种规划的 15 岁以上人群比较分析的文章。≥我们将规划分为普遍型和针对母亲乙肝表面抗原 (HBsAg) 呈阳性的婴儿。我们纳入了乙肝核心抗体 (HBcAb) 患病率或乙肝表面抗原 (HBsAg) 状态或两者皆有的研究。我们评估了研究方法质量并估计感染患病率的相对约简。

**结果:**

其中，有 26 项研究符合纳入标准，绝大多数研究来自中国（20 项研究）。在未接种疫苗的人群和接种普遍型疫苗的人群中，乙肝病毒 (HBV) 感染患病率分别在 0.6%(116/20305)和 16.3%(60/367) 之间与 0.3%(1/300) 和 8.5%(73/857) 之间。与未接种疫苗的人群相比，接种普遍型疫苗的人群的相对患病率为乙肝表面抗原 (HBsAg) 0.24%（95% 置信区间，CI：0.16-0.35） ，乙肝核心抗体 (HBcAb)0.23(95% CI： 0.17-0.32) 。对于有针对性疫苗接种的人群，相对患病率分别为 0.32(95% CI：0.24-0.43 )和 0.33(95% CI：0.23-2.3) 。

**结论:**

若干人群中接种疫苗的剩余负担表明需要对疫苗接种覆盖率、及时性和方案质量的其他方面进行比较长期的评估。随着接种乙肝病毒 (HBV) 疫苗的婴儿群体进入成年期，对青少年和年轻人患病率的持续分析将确认消除乙肝的努力步入正轨。****

## Резюме

**Цель:**

Провести систематический обзор и метаанализ долгосрочного влияния вакцинации младенцев на распространенность вирусного гепатита В (HBV) на уровне популяции.

**Методы:**

В онлайн-базах данных авторы провели поиск статей, в которых сообщается о сопоставлении популяционных когорт, включающих детей в возрасте ≥□15 лет, которые ранее были иммунизированы в рамках программ иммунизации младенцев против HBV либо не иммунизированы. Авторы классифицировали программы как универсальные либо направленные на младенцев, у матерей которых был обнаружен положительный результат анализа на поверхностный антиген вируса гепатита В (HBsAg). Авторы также включили исследования, в которых сообщалось о распространенности положительного результата анализа на антитела к капсидному антигену вируса гепатита В (HBcAb) или статуса по HBsAg либо обоих показателей. Была проведена оценка качества методов исследования и относительного снижения распространенности инфекции.

**Результаты:**

Из 26 исследований, которые соответствовали критериям включения, большинство было проведено в Китае (20 исследований). Распространенность HBV в невакцинированных и универсально вакцинированных когортах варьировала от 0,6% (116 из 20 305 человек) до 16,3% (60/367) и от 0,3% (1/300) до 8,5% (73/857) соответственно. При сравнении когорт с универсальной вакцинацией и без вакцинации относительная распространенность составила 0,24 (95%-й ДИ: 0,16–0,35) для HBsAg и 0,23 (95%-й ДИ: 0,17–0,32) для HBcAb. В популяциях с целенаправленной вакцинацией относительная распространенность составила 0,32 (95%-й ДИ: 0,24–0,43) и 0,33 (95%-й ДИ: 0,23–0,45) соответственно.

**Вывод:**

Остаточное бремя инфекции в нескольких когортах, которым была проведена вакцинация, свидетельствует о том, что необходимы долгосрочные оценки охвата вакцинацией, временного охвата и других аспектов качества программы. По мере взросления вакцинированных от HBV когорт младенцев постоянный анализ распространенности заболевания среди подростков и молодых людей обеспечит успех усилий по ликвидации этой инфекции.****

## Introduction

Infection with the hepatitis B virus (HBV) is a major global cause of ill health that particularly affects low- and middle-income countries. An estimated 257 million people have chronic HBV infection, and 686 000 deaths occur annually due to long-term complications including liver cirrhosis and hepatocellular carcinoma, a number that is projected to increase.^1^^,^^2^ Most chronic infection is acquired in infancy or early childhood, primarily through mother-to-child transmission.^3^^,^^4^ Exposure later in life, through sexual intercourse or blood contact, can also lead to chronic infection but more frequently results in viral clearance and immunity.

Vaccines against HBV infection are highly effective. A plasma-derived vaccine, first used in a national infant immunization programme in 1984,^5^ was gradually replaced by recombinant vaccines, which can be manufactured at greater scale.^6^ The global recommended schedule is a dose at birth, ideally within 24 hours, followed by two to three doses at monthly intervals.^7^

A few countries have reported declines in the prevalence of HBV infection following the implementation of infant vaccination programmes,^8^^,^^9^ and declining incidence of liver cancer in children and young adults.^10^^–^^14^ The World Health Organization’s (WHO’s) Member States aspire to the goal of global elimination of HBV infection and its consequences, with the central strategy being infant vaccination programmes to achieve direct and herd protection.^15^ WHO has set an elimination target of a 90% reduction in prevalence by 2030,^16^ and specified two primary target indicators: cumulated incidence of HBV infection in children aged 5 years; and deaths from hepatocellular carcinoma, cirrhosis and chronic liver diseases attributable to HBV infection.^17^

Although these two indicators effectively represent programme goals, neither are straightforward to monitor in a consistent way over time. Incidence in 5-year-olds is estimated through surveys that require the collection of specimens from a representative sample, which can be difficult to obtain in this age group.^9^ Cause-specific mortality is difficult to measure reliably, particularly in countries with constrained resources. It could take decades for mortality data to fully reflect vaccine-related improvements, due to the long latency of chronic HBV infection in the causation of liver disease.

Prevalence of infection in the wider population is more straightforward to measure and is an earlier and more specific indicator of impact of immunization programmes than measuring prevalence in 5-year-olds alone. Population prevalence is also the core indicator recommended as the first priority by WHO.^17^ With infant vaccination now in place for several decades in several countries, it is timely to consider how well it is achieving reductions in HBV infection. This is particularly important as vaccinated generations enter adulthood, a period of higher risk of exposure through sexual activity,^18^ and become the potential source of transmission to the next generation. We therefore conducted a systematic review and meta-analysis of studies assessing changes in the prevalence of HBV markers in populations offered universal or targeted infant immunization at least 15 years before.

## Methods

This review was registered and conducted according to the Preferred Reporting Items for Systematic Reviews and Meta-Analyses guidelines. Registration is available at: http://www.crd.york.ac.uk/PROSPERO/display_record.asp?ID=CRD42017060309 (the checklist is available from the corresponding author).

### Search strategy

We searched the online databases of Embase®, MEDLINE®, Web of Science and LILACS to January 2017. The search terms used were: “hepatitis B” AND (“vaccination” OR “mass vaccination” OR “immunization” OR “immunization programmes”) AND (“adolescent” OR “adult”). The search was supplemented with manual searches of reference lists of articles for additional studies.

### Inclusion criteria

We included any study addressing the long-term impact of an infant hepatitis B vaccine programme at a population level if it satisfied the following criteria: (i) HBV infection status, as defined by hepatitis B core antibody (HBcAb) or hepatitis B surface antigen (HBsAg) status (or both), was assessed and reported in the study population; (ii) the study population, or a specified subgroup for which data were reported separately, was aged ≥ 15 years at the time when HBV infection status was assessed; (iii) the study population, or a specified subgroup meeting criterion ii, included a cohort not offered vaccination through an infant programme (referred to here as the unvaccinated cohort); and (iv) the study population, or a specified subgroup meeting criterion ii, included a cohort offered vaccination through an infant programme (referred to as the vaccinated cohort).

We categorized infant vaccination programmes as either universal, if hepatitis B vaccine was reported as being available to all newborns; or targeted, if vaccine was available to infants born to women screening positive for chronic HBV infection or to women at high risk for some other reason. In some countries, catch-up vaccination programmes were made available to children born a few years before implementation of universal infant vaccination. In these settings, cohorts classified as targeted may have been involved in both a targeted vaccination programme and a catch-up programme.

The primary outcomes of interest were the serological prevalences of (i) HBsAg, which defines chronic HBV infection, and (ii) HBcAb, which defines past, cleared HBV infection in people who are negative for HBsAg.

Two authors independently reviewed the abstracts of the studies identified by the search strategy for studies that met the inclusion criteria. Chinese language articles were translated by a third author to determine if they met the inclusion criteria. The full texts of articles that appeared to be relevant were reviewed by the two authors independently for inclusion. If the same cohort of individuals appeared to have been included in more than one publication, we contacted the authors of the articles to define overlap and avoid duplication.

### Variables extracted

We extracted the following study variables for each eligible study, if available: study design, location (country and region); study period; participants’ age and sex distribution; vaccine programme coverage as reported in the article, including coverage of timely birth dose; number of participants in unvaccinated and vaccinated cohorts; and the number positive for HBsAg and/or HBcAb by serology testing in each cohort. We contacted the authors of the articles if data were unavailable in the original publication.

### Quality assessment

In addition to data extraction, two authors separately reviewed the quality of each study using an adapted Cochrane method.^19^ We considered whether studies had addressed potential confounding variables, used a repeatable sampling frame and calculated a response rate. We also judged whether there was potential for bias through assessment of the serological markers, selective reporting of data or the representativeness of populations surveyed.^19^

### Statistical analyses

We classified studies according to outcome measures (prevalence of HBsAg, HBcAb or both) and whether they reported on universal or targeted infant vaccination programmes. We calculated the relative prevalence (RP) and corresponding 95% confidence intervals (CI) as the ratio of the prevalence in vaccinated and unvaccinated cohorts within each study. Meta-analyses were performed by pooling across studies using the Mantel‒Haenszel method. We assessed statistical heterogeneity using *I^2^*, with a value of >  30% as the cut-off.^20^ A fixed-effects model was used when there was no significant heterogeneity and a random-effects model otherwise. Publication bias was evaluated visually with a funnel plot. We also conducted separate meta-analyses restricted to studies in which the comparisons involved subjects of the same age, and to determine if effects differed by HBsAg prevalence (< 10% versus ≥ 10%) in the unvaccinated cohort or by geographical location. All analyses were conducted using RevMan version 5.3 (Review Manager, The Cochrane Collaboration, Copenhagen, Denmark).

## Results

**Fig. 1:**
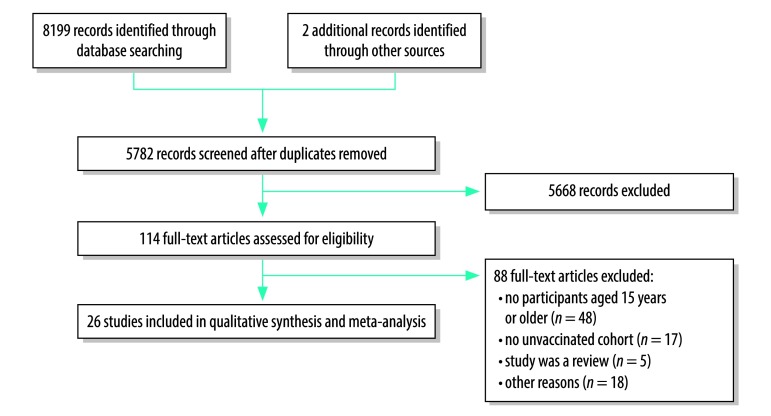
Flowchart of selection of studies for the meta-analysis of the long-term impact of hepatitis B virus immunization programmes

The electronic search yielded 5781 unique articles which we screened for inclusion (Fig. 1). Of these, we assessed 114 full-text articles for eligibility and excluded 88 that were ineligible for various reasons. As three publications^21^^–^^23^ reported on the same subject group in overlapping study periods, only data from the most recent article was included.^21^

### Study characteristics

Of the 26 studies that met the inclusion criteria (Table 1)^21^^,^^24^^–^^48^ most were from Taiwan, China (14 articles),^21^^,^^24^^–^^26^^,^^28^^,^^29^^,^^31^^–^^35^^,^^37^^,^^39^^,^^47^ six from mainland China,^36^^,^^40^^,^^41^^,^^44^^,^^46^^,^^48^ two each from the Gambia^30^^,^^43^ and Italy,^27^^,^^42^ and one each from Australia^38^ and Fiji.^45^ The number of study participants aged 15 years or older varied substantially, from 259^37^ to 738 195^40^ (median: 3776). Eleven studies included both targeted and universal vaccination cohorts,^21^^,^^28^^,^^29^^,^^31^^,^^32^^,^^34^^,^^35^^,^^37^^–^^39^^,^^47^ four studies included a targeted vaccination cohort only,^24^^–^^26^^,^^41^ while 11 studies had only a universal vaccination cohort.^27^^,^^30^^,^^33^^,^^36^^,^^40^^,^^42^^–^^46^^,^^48^ In virtually all studies, unvaccinated cohorts were born in years before the implementation of the newborn vaccination programme or had passed the qualifying age at the time of implementation of catch-up programmes (or both).

**Table 1:** Summary of studies included in the meta-analysis of the long-term impact of hepatitis B virus immunization programmes

Reference	Location	Population	Study design	Study period	Study sample, no.	Sex, %	Vaccination programme	Age of cohorts, years	HBsAg prevalence, %, by vaccination cohort^a^		
									Unvaccinated	Targeted	Universal
Lin et al., 2003^24^	Hualien and Eastern Taiwan, China	High-school students	Repeated cross-sectional seroprevalence surveys	1991–2001	10 194	M: 54; F: 46	Targeted	Unvaccinated: 15; targeted: 15	12.1	4.1	NA
Chang et al., 2007^25^	Taipei, China	Students entering university	Cross-sectional seroprevalence survey	2003–2004	7 592	M: 53; F: 47	Targeted	Overall mean: 19.8 (range: 16.1–54.2)	7.4	2.2	NA
Chen et al., 2007^26^	Central Taiwan, China	Medical students attending university	Cross-sectional seroprevalence survey	2000–2003	4 575	M: 47; F: 53	Targeted	Overall mean: 18.7 (range: 17–38)	8.0	3.6	NA
Da Villa et al., 2007^27^	Afragola, Italy	Residents of Afragola, Italy	Repeated cross-sectional seroprevalence surveys	1978 and 2006	660**^b^**	M: 51; F: 49	Universal	Unvaccinated: 15–20; universal: 15–20	10.3	NA	0.3
Ni et al., 2007^28^	Taipei city, Taiwan, China	Participants recruited from schools, institutes, or workplaces	Cross-sectional seroprevalence survey	2004	1 142	M; F^c^	Targeted, Universal	Unvaccinated: 20–30; targeted: 18–19; universal: 15–17	10.9	2.1	1.5
Su et al., 2007^29^	Northern Taiwan, China	New entry university students	Cross-sectional seroprevalence survey	2005	1 969	M; F^d^	Targeted, universal	Unvaccinated: 21+	8.7	3.2	1.7
								Targeted: 19–21; universal: 17–19			
Van der Sande et al., 2007^30^	Gambia^e^	Participants in the Gambia Hepatitis Intervention Study	Cross-sectional seroprevalence survey	2004	1 000	M: 45; F: 55	Universal	Unvaccinated: 15; universal: 15	12.1	NA	0.5
Lin et al., 2008^31^	Southern Taiwan, China	Pregnant Taiwanese women receiving prenatal examinations	Cross-sectional seroprevalence survey	1996–2005	10 327	F: 100	Targeted, universal	Not reported	15.7	11.4	3.1
Lu et al., 2009^32^	Central and northern Taiwan, China	College and private university students	Repeated cross-sectional seroprevalence surveys	2000–2007	4 193	M: 28; F: 72^f^	Targeted, universal	Unvaccinated: 18; targeted: 15–18; universal: 15–18	11.6	3.5	1.15
Sun et al., 2009^33^	Taiwan, China	Consecutive HIV-negative persons seeking health check-up	Cross-sectional seroprevalence survey	2004–2007	2 594^h^	M: 69; F: 31	Universal	Overall median: 38 (range: 16–94)	15.5	NA	8.5
Chen et al., 2011^21^	21 universities across Taiwan, China	New entry university students	Repeated cross-sectional seroprevalence surveys	1995–2009	101 584	M: 53; F: 47	Targeted, universal	Overall mean: 18.5 (range: 17.8–20.7)	11.8	2.3	1.9
Chu et al., 2011^34^	Northern Taiwan, China	Clinic attendees and university students	Cross-sectional seroprevalence survey	2008	2 515	M: 60; F: 40	Targeted, universal	Unvaccinated, mean: 41.1 targeted, mean: 22.8; universal, mean: 18.6	16.3	5.2	2.8
Lin et al., 2011^35^	Central Taiwan, China	Undergraduate and graduate students entering university	Cross-sectional seroprevalence survey	2005	1 677	M; F^c^	Targeted, universal	Unvaccinated: 21+; targeted: 19–21; universal: 17–19	11.7	1.6	1.7
Shen et al., 2011^36^	Long An county, China	Residents of five villages in Long An county	Cross-sectional seroprevalence survey	2005	3 410	M; F	Universal	Unvaccinated: 20–94; universal: 15–19	7.1	NA	5.5
Lai et al., 2012^37^	Northern, central, southern and eastern Taiwan, China	Participants recruited into epidemiology study for vaccine-preventable diseases	Cross-sectional seroprevalence survey	2007	259	M; F^c^	Targeted, universal	Unvaccinated: 23+; targeted: 21–23; universal: 18–21	9.3	9.4	2.0
Liu et al., 2012^38^	Northern Territory, Australia	Aboriginal women giving birth in public hospitals	Cross-sectional seroprevalence survey	2005–2010	5 678	F: 100	Targeted, universal	Unvaccinated mean: 27.2; universal mean: 18.0	3.5	2.2	0.8
Ni et al., 2012^39^	Taipei city, Taiwan, China	Participants recruited from schools, institutes or workplaces	Cross-sectional seroprevalence survey	2009	1 681^f^	M; F^c^	Targeted, universal	Unvaccinated: 26–29; targeted: 24–25; universal: 15–23	8.2	4.5	1.2
Yang et al., 2012^40^	Zhejiang province, China	Participants of province-wide health examination plan	Cross-sectional seroprevalence survey	2010	738 195	M: 41; F: 59^g^	Universal	Unvaccinated: 20+; universal: 15–19^i^	7.2	NA	3.5
Zhang et al., 2012^41^	Shanghai, China	Infants born in 1986 from Huang Pu district, Shanghai	Cross-sectional seroprevalence survey	2007–2009	1 204	M; F^d^	Targeted	21–30	14.2	0.6	NA
Boccalini et al., 2013^42^	Tuscany, Central Italy	Hospital outpatients in Tuscany	Cross-sectional seroprevalence study	2009	762	M: 50; F: 50	Universal	Unvaccinated: 31–50; universal: 21–30	NA^j^	NA^j^	NA^j^
Peto et al., 2014^43^	Gambia	Participants of the Gambia Hepatitis Intervention Study born 1986–1990	Per-protocol analysis of cluster randomized trial	2007–2008	753	M; F^d^	Universal	Unvaccinated: 17–22; universal: 17–22	12.4	NA	2.2
Liao et al., 2014^44^	Guangxi Zhuang autonomous region, China	Students from one college in Liuzhou city	Cross-sectional seroprevalence study	2009	392	M: 18; F: 82	Universal	Unvaccinated: 31–50; universal: 21–30	12.0	NA	5.3
Tsukakoshi et al., 2015^45^	Fiji	Residents of the central, western and northern health divisions of Fiji	Cross-sectional seroprevalence study	2008–2009	504	M: 80^k^; F: 20	Universal	Unvaccinated: 21–49; universal: 16–20	3.2	NA	5.7
Chen et al., 2016^46^	Qidong, China	Participants of the Qidong Hepatitis B Intervention Study	Per-protocol analysis of cluster randomized trial	2013	8 301	M; F^d^	Universal	Unvaccinated, mean: 26.5; universal, mean: 25.6	9.0	NA	2.4
Ni et al., 2016^47^	Taipei, Taiwan, China	Participants recruited from schools, institutions and workplaces	Cross-sectional seroprevalence survey	2014	3 036	M; F^c^	Targeted, universal	Unvaccinated: 30–50; universal: 15–29	7.0	3.2	0.6
Wang et al., 2016^48^	Shenzhen, China	Blood donors	Cross-sectional seroprevalence study	2005–2014	118 423	M: 67; F: 33	Universal	Unvaccinated: 18–22^l;^ universal: 18–22^l^	3.89^m^; 0.57^n^	NA	3.51^m^; 0.27^n^

F: female; HBV: hepatitis B virus; HBsAg: hepatitis B surface antigen; HIV: human immunodeficiency virus; M: male; NA not applicable.

^a^ Targeted refers to infant vaccination programmes in which vaccine was available to infants born to women found on the basis of screening to have chronic HBV infection or determined to be at high risk for some other reason. Universal refers to infant vaccination programmes in which hepatitis B vaccine was reported as being available to all newborns. Unvaccinated refers to cohorts that had no universal or targeted hepatitis B immunization programmes.

^b^ We only included 15–20 years age group from this study.

^c^ Sex distribution not reported in the article for ages ≥ 15 years.

^d^ Sex distribution not reported in the article.

^e^ Region not reported in the article.

^f^ Total sex distribution for participants aged ≥ 15 years.

^g^ Age distribution for all participants included in the article, aged ≥ 0 years.

^h^ We excluded HIV-positive participants from total and calculations.

^i^ Data for ages 15–19 years was requested and provided by authors (article only provides data for 0–20 year age group collectively).

^j^ Article reported anti-hepatitis core antibody levels only.

^k^ Sex distribution only reported in the article for vaccinated group, age 21–49 years.

^l^ Age range for both first-time and repeat blood donor groups.

^m^ First-time blood donors.

^n^ Repeat blood donors.

Twenty studies used a single cross-sectional seroprevalence survey design,^23^^,^^25^^,^^26^^,^^28^^–^^31^^,^^33^^–^^42^^,^^44^^,^^45^^,^^47^^,^^48^ four involved two or more cross-sectional seroprevalence survey time points^21^^,^^24^^,^^27^^,^^32^ and two were per-protocol analyses of a cluster randomized trial.^43^^,^^46^ Participants included university entrants,^21^^,^^25^^,26.47,^^35^^,^^44^ high-school students,^24^ women giving birth,^31^^,^^38^ blood donors,^48^ clinic attendees seeking a health check-up,^33^ hospital outpatients,^42^ members of population-based cohorts^27^^,^^30^^,^^37^^,^^41^^,^^43^^,^^46^ and participants in health screening.^40^ Some studies involved participants from more than one source.^28^^,^^32^^,^^34^^,^^36^^,^^39^^,^^45^^,^^47^

### Study quality

Few studies reported on participation rates for serological testing in the target populations. Those that did were conducted in institutional settings and participation rates were very high. Two studies restricted recruitment to those whose immunization history was recorded.^30^^,^^43^ Potentially confounding variables were reported in a minority of studies, and were generally confined to age, sex and region. The sampling method appeared to be repeatable in studies based in institutions or on recruitment of blood donors, but was not clear for the other studies. There was no evidence of bias in the strategies used for serological testing or in the data selected for presentation in reports. Generalizability to wider regional or national populations was not addressed in any of the studies.

### Prevalence of HBV markers

**Fig. 2:**
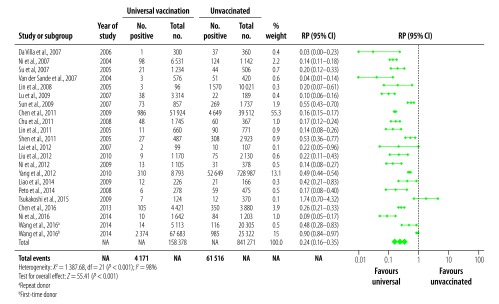
Relative prevalence of hepatitis B surface antigen in the meta-analysis of the long-term impact of immunization programmes: comparison of universal vaccination and unvaccinated cohorts

CI: confidence interval; df: degrees of freedom; NA: not applicable; RP: relative prevalence.

^a^ Repeat donor.

^b^ First-time donor.

Notes: The figure shows the number of participants positive for the hepatitis B surface antigen and the total number of participants in the cohort being measured. Totals may not equal the total number of participants in Table 1, as only age-eligible participants were included in our analysis.

**Fig. 3:**
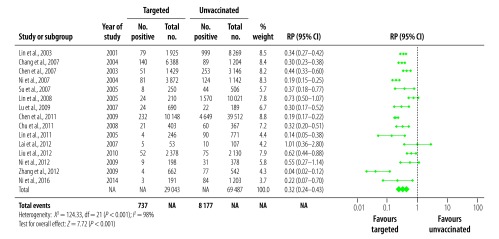
Relative prevalence of hepatitis B surface antigen in the meta-analysis of the long-term impact of immunization programmes: comparison of targeted vaccination and unvaccinated cohorts

CI: confidence interval; df: degrees of freedom; NA: not applicable; RP: relative prevalence.

Notes: The figure shows the number of participants positive for the hepatitis B surface antigen and the total number of participants in the cohort being measured. Totals may not equal the total number of participants in Table 1, as only age-eligible participants were included in our analysis.

We analysed 21 studies reporting on HBsAg prevalence in populations exposed to universal vaccination compared with those given no vaccination (Fig. 2). The prevalence in the universally vaccinated cohorts ranged from 0.3%^27^ to 8.5%^33^ (median: 2.0%). The prevalence of HBsAg in the corresponding no vaccination cohorts were substantially higher, ranging from 0.6%^48^ to 16.3%^34^ (median: 9.8%). All except one study^45^ reported a decrease in HBsAg prevalence; the combined RP in vaccinated populations was 0.24 (95% CI: 0.16–0.35; Fig. 2). Among 15 studies that reported on HBsAg in a targeted vaccination cohort (Fig. 3), prevalences ranged from 0.6%^41^ to 11.4%^31^ (median: 3.2%), compared with a range of 3.5%^38^ to 16.3%^34^ (median: 10.9%) in the corresponding no vaccination cohorts, leading to a combined RP of 0.32 (95% CI: 0.24–0.43; Fig. 3). Highly significant statistical heterogeneity between studies was found in the meta-analyses of HBsAg prevalence in studies comparing both universal and targeted vaccination with unvaccinated cohorts (*I^2^* = 98%, *P* < 0.001 and *I^2^* = 89%, *P* < 0.001, respectively). We therefore used random-effects models to estimate combined RPs and CIs.

**Fig. 4:**
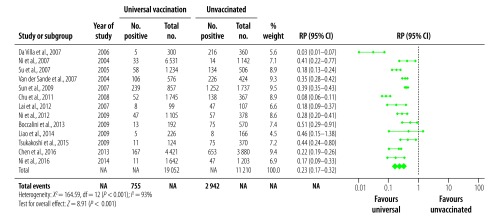
Relative prevalence of hepatitis B core antibody in the meta-analysis of the long-term impact of immunization programmes: comparison of universal vaccination and unvaccinated cohorts

CI: confidence interval; df: degrees of freedom; NA: not applicable; RP: relative prevalence.

Notes: The figure shows the number of participants positive for the hepatitis B surface antigen and the total number of participants in the cohort being measured. Totals may not equal the total number of participants in Table 1, as only age-eligible participants were included in our analysis.

**Fig. 5:**
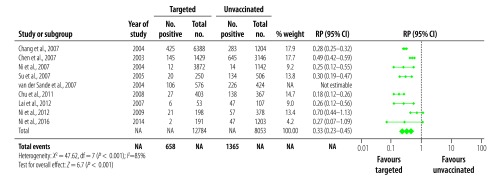
Relative prevalence of hepatitis B core antibody in the meta-analysis of the long-term impact of immunization programmes: comparison of targeted vaccination and unvaccinated cohorts

CI: confidence interval; df: degrees of freedom; NA: not applicable; RP: relative prevalence

Notes: The figure shows the number of participants positive for the hepatitis B surface antigen and the total number of participants in the cohort being measured. Totals may not equal the total number of participants in Table 1, as only age-eligible participants were included in our analysis.

For the corresponding analyses of HBcAb prevalence, there were 13 studies that reported data from a universal vaccination cohort (Fig. 4), and nine that reported on targeted vaccination (Fig. 5). The prevalence of HBcAb in the corresponding no vaccination cohorts ranged from 1.2%^28^ to 72.1%^33^ (median: 20.3%) and from 1.2%^28^ to 43.9%^37^ (median: 22.0%), respectively. In vaccinated cohorts, prevalences were between 0.5%^28^ to 27.9%^33^ (median: 4.3%) for universal vaccination cohorts and 0.3%^28^ to 11.3%^37^ (median: 7.4%) in targeted vaccination cohorts. The summary RPs for HBcAb prevalence were 0.23 (95% CI: 0.17–0.32) for universal and 0.33 (95% CI: 0.23–0.45) for targeted vaccination. As with the HBsAg analyses, we used random-effect models for estimates and CI due to the highly significant heterogeneity.

**Fig. 6:**
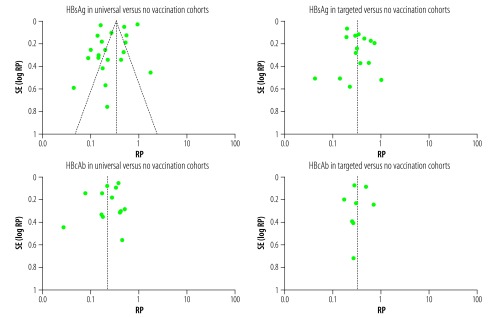
Funnel plot of publication bias in studies of the prevalence of hepatitis B virus markers in adults

Funnel plots for each of the four analyses were symmetrical around the combined RPs (Fig. 6).

Hepatitis B core antibody: HBcAb; HBsAg: hepatitis B surface antigen; RP: relative prevalence; SE: standard error.

Notes: The relative prevalence is shown. Visual examination of the plot reveals symmetry of the individual studies to the combined prevalence ratio for the 22 studies.

### Coverage

A birth dose of vaccine, either within 24 hours or at birth, was included in the vaccination schedule for all studies except one from Italy,^27^ where birth dose was only included in the schedule for targeted vaccination. The main parameter used for coverage was administration of three doses of HBV vaccine. There was limited information on coverage for vaccinated cohorts. Five studies used self-reporting, and found coverage ranging from 55.9% (7 700 of 13 765 people) to over 98% (872/878 people).^25^^,^^28^^,^^36^^,^^37^^,^^45^ Two cohort studies were able to verify the vaccination status of the entire vaccinated group.^30^^,^^43^ One study reported 76.1% (842/1107 people) coverage of the third vaccine dose overall, but 34.5% (35/99 people) for those older than 15 years in the universal vaccination cohort and 19.0.% (10/53 people) in the targeted vaccination cohort.^37^

Only one study reported on timeliness of the birth dose, stating that 65 of 215 participants (30.2%) who had received the full schedule received their first dose within 24 hours.^44^

While some studies reported on coverage at a national or regional level, coverage was not always specified for the age group included in the review and numerators and denominators were not stated. Overall coverage of universal vaccination in Taiwan, China, as cited by some studies, was generally high, ranging from 86.9 to 98.0%.^24^^,^^28^^,^^31^^–^^33^^,^^39^^,^^47^ In Italy coverage was cited in the paper to have been 63% in 1991 increasing to 99% in some regions by 1996.^42^ In mainland China, studies cited coverage ranging from 30% in the earlier years of the vaccination programme to 99.9% in 2010.^40^^,^^48^ In the Australian study, coverage among residents of remote indigenous communities was cited to be 90%.^38^

### Sub-group analyses 

**Fig. 7:**
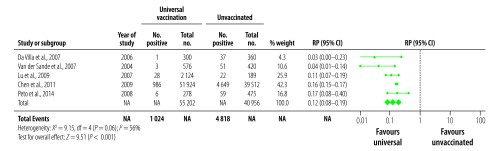
Relative prevalence of hepatitis B surface antigen in the meta-analysis of the long-term impact of immunization programmes: comparison of targeted vaccination and unvaccinated cohorts with participants in the same age group

CI: confidence interval; df: degrees of freedom; NA: not applicable; RP: relative prevalence.

Notes: The figure shows the number of participants positive for the hepatitis B surface antigen and the total number of participants in the cohort being measured. Totals may not equal the total number of participants in Table 1, as only age-eligible participants were included in our analysis. For the study of Lu et al., 2009 we included only 18-years-olds in this analysis. 15-year-olds were excluded as this group received universal vaccination.

We also restricted analysis to the five studies that compared HBsAg prevalence between universally vaccinated and unvaccinated cohorts of the same age (Fig. 7).^21^^,^^27^^,^^30^^,^^32^^,^^43^ The combined RP was 0.12 (95% CI: 0.08–0.19) compared with 0.30 (95% CI: 0.21–0.43) in 16 studies based on comparisons of cohorts at different ages.^28^^,^^29^^,^^31^^,^^33^^–^^40^^,^^44^^–^^48^

Restricting analyses to those with HBsAg prevalence under 10% in the unvaccinated group yielded 10 studies, all of which assessed HBsAg positivity in cohorts with universal vaccination.^29^^,^^30^^,^^36^^–^^40^^,^^45^^,^^47^^,^^48^ The combined RP was 0.34 (95% CI: 0.23–0.52). In comparison, RP was 0.17 (95% CI: 0.12–0.25) across the 11 studies with HBsAg prevalence of at least 10% in the unvaccinated cohort.^21^^,^^27^^,^^28^^,^^30^^–^^35^^,^^43^^,^^44^ For targeted vaccination cohorts, the RP based on seven studies with HBsAg prevalence under 10% in the corresponding unvaccinated cohorts was 0.42 (95% CI: 0.32–0.56).^25^^,^^26^^,^^29^^,^^37^^–^^39^^,^^47^ It was 0.24 (95% CI: 0.16–0.36) in eight studies for which prevalence was above 10% in the unvaccinated cohorts.^24^^,^^28^^,^^31^^,^^32^^,^^34^^,^^35^^,^^37^^,^^41^

Restricting analyses to studies from Taiwan, China (11 studies),^21^^,^^28^^,^^29^^,^^31^^–^^35^^,^^37^^,^^39^^,^^47^ for universal vaccination cohorts, the combined RP for protection was 0.17 (95% CI: 0.12–0.24), while for the 10 studies from other countries^27^^,^^30^^,^^36^^,^^38^^,^^40^^,^^43^^–^^46^^,^^48^ the reduction in risk was 0.36 (95% CI: 0.24–0.54). For targeted vaccination, the RP was 0.33 (95% CI: 0∙.25–0.44) for studies from Taiwan, China (13 studies),^21^^,^^24^^–^^26^^,^^28^^,^^29^^,^^31^^,^^32^^,^^34^^,^^35^^,^^37^^,^^39^^,^^47^ with only two such studies from another area.^38^^,^^41^

## Discussion

Our meta-analysis of the long-term impact on infection of infant hepatitis B vaccination programmes at the population level focused on long-term impact by restricting the time period to at least 15 years following vaccination. An earlier review of impact did not report a combined estimate and did not specifically focus on long-term impact.^49^ We found that adolescents and adults in birth cohorts that were offered universal infant vaccination had a 76% lower prevalence of HBV infection and a similar reduction in risk (77%) of HBcAb prevalence compared with cohorts for whom infant vaccine programmes were unavailable. The effect was similar for both higher and lower levels of HBsAg prevalence in the unvaccinated population. The impact of targeted vaccination programmes was slightly lower (68% and 67%, respectively). As a result of overlap in the relevant cohorts, it was not possible to distinguish the effect of these programmes from the impact of catch-up programmes. It was also not possible to separate the direct effect of targeted programmes from herd effects of reduced horizontal transmission, both in vaccinated same-age peers and slightly younger universally vaccinated cohorts.

Our findings are valuable for demonstrating in a systematic way what extent of reductions in prevalence could be expected from infant HBV vaccination programmes. Most countries (184 countries in 2014)^50^ have implemented such programmes, but few have so far reported on long-term impact. As cohorts of adolescents and young adults are entering a period of exposure risk through sexual activity, and will become parents of the next generation, it is appropriate to measure prevalence of infection in this age group.^18^

The similarity between the impact on HBcAb and HBsAg suggests that the vaccine’s mechanism of protection applies equally to preventing infection and stopping it from becoming established. Nevertheless, there was still evidence of acquisition of HBV infection in all of the vaccine-eligible populations. The most likely explanation for residual infection is incomplete vaccine coverage or non-timely vaccination.^28^^,^^37^^,^^42^^,^^45^

 Studies have shown that perinatal HBV transmission is higher in infants who received the birth dose late or received fewer than the three scheduled doses.^51^^,^^52^ Global coverage of the full HBV vaccination schedule is estimated at 84%,^50^ while administration of the birth dose remains low at an estimated 39% in 2015.^4^ The few studies in our systematic review that reported on vaccine coverage recorded highly varying rates. Most of the vaccinated cohorts from the studies in Taiwan, China, were born in the early years of the infant vaccination programme when coverage was lower. The only study reporting on administration of the birth dose within 24 hours, from mainland China, found coverage of 30.2%.^44^ No studies reported on the actual timing of the birth dose. Lack of timeliness of the birth dose and inadequate coverage of the full schedule are likely to have contributed to the residual infection that we found in vaccinated cohorts. Further monitoring of more recent cohorts, including reporting of timeliness of the birth dose, is needed to determine the impact of improved coverage on infection rates.

Vaccine failure, primary (no initial immune response) or secondary (waning of immunity), may have been another cause of infections in the vaccinated cohorts.^53^ Poor immunological response was demonstrated in reports of both active and cleared HBV infection detected among fully vaccinated people.^54^^–^^57^ HBV prevalence in vaccinated populations could also be attributed to mutations in the HBV S-gene, which can allow the virus to avoid being neutralized by the vaccine-generated anitbodies.^53^^,^^58^ However, while they need to be monitored, these mutants probably have little significance at a public health level and are unlikely to be a major cause of prevalence in vaccinated cohorts.^58^

More than half of the studies included in this meta-analysis were from Taiwan, China, reflecting a strong, early commitment to HBV prevention and research in this formerly high-prevalence area. The reduction in HBsAg prevalence was markedly higher for these studies than those in other areas (83% versus 64%). The early attainment of high newborn coverage of hepatitis B vaccine in Taiwan, China, over 95% by 2002,^11^ could explain this observation.

The studies we included had limitations. Most were based on a single time-point, and compared people who had received vaccination with those who had not, on the basis of their birth cohort. As these two groups were inevitably of different ages at the time of surveying, it is not possible to remove any effect of age from the comparison. However, it is likely that most chronic infections were acquired in infancy and early childhood, and that prevalence would have been relatively stable in adolescence and adulthood, with at most a slight age-related increase. On the other hand, the estimated reduction in prevalence was 88% for the few studies that compared cohorts of the same age, as opposed to 70% in the single time-point surveys. This finding was surprising, as we might expect that studies comparing younger vaccinees to older unvaccinated cohorts would generate a more favourable estimate if there had been any increase of prevalence with age. However, comparisons involving same-age cohorts may have been affected by other, unknown confounders.

Limitations of the meta-analysis included the considerable heterogeneity in estimated effect sizes, and the statistically dominant role played by a small number of studies that contributed large numbers. Heterogeneity may have been due to differences in study designs, populations and immunization coverage, and to the characteristics identified in our assessment of study quality, such as response rates. Nevertheless all studies except one reported substantially lower HBsAg prevalence in the vaccinated cohort compared with the unvaccinated group, and the one exception reported low coverage in the vaccinated cohort.^45^

Another issue to consider in interpreting the findings is the relatively narrow geographical spread of studies. Although many countries have had historically high prevalence of hepatitis B, particularly in the WHO Western Pacific and African regions,^59^ most of the published studies that met our inclusion requirements were from China. Few studies from Africa met the inclusion criteria, so we were unable to separately assess impact in this region, where horizontal transmission is believed to play a greater role of transmission compared with Asian countries where perinatal transmission dominates.^60^ Determining the impact of vaccination in African countries would be important, particularly the impact of timely birth dose vaccination on reductions in prevalence in this setting. 

With infant HBV vaccination programmes now widely in place, many countries could now evaluate the longer term impact on the prevalence of infection. The ideal mechanism for this evaluation is a time-series comparison with observations at regular time-points, from comparable populations. In many countries, an efficient approach is serological testing done routinely in pregnant women or other populations, such as new entrants to university, the military or other institutions. All these populations are likely to be accessible for routine, repeatable monitoring. As identified in our quality assessment, such designs should incorporate repeatable sampling frames, measure participation rates and adjust for potential confounding variables. While prevalence in 5-year-olds provides a more immediate indicator of impact, large representative samples in this age group cannot be easily accessed in many settings.

Our estimated reduction in prevalence is consistent with findings of modelling studies which suggested that routine infant vaccination from birth with global coverage of 90% could prevent 4.3 million new infections from 2015 to 2030,^61^ and could prevent 84% of HBV-related deaths in the hypothetical birth cohort from the year 2000.^61^^,^^62^ Our findings strongly support the importance of the WHO target of 90% coverage of the hepatitis B vaccine in infancy,^15^ as low coverage is a potential contributor to residual HBV prevalence. The evidence presented here shows that elimination is achievable, but still on the far horizon. Analyses of available serological data on HBV prevalence in adolescents and young adults can provide information on gaps in the pathway to elimination that can then be addressed through programmatic measures.
